# CTA-based classification of occipital artery course variations at the atlanto-occipital interval, with surgical implications

**DOI:** 10.1007/s00276-026-03853-7

**Published:** 2026-03-05

**Authors:** Viviana Dincă, Mugurel Constantin Rusu, Sorin Hostiuc, Adelina Maria Jianu

**Affiliations:** 1 Division of Anatomy, Department 1, Faculty of Dentistry, “Carol Davila” University of Medicine and Pharmacy, 8 Eroilor Sanitari Blvd., 050474 Bucharest, Romania; 2 Division of Legal Medicine and Bioethics, Faculty of Dentistry, “Carol Davila” University of Medicine and Pharmacy, 050474 Bucharest, Romania; 3 Department of Anatomy and Embryology, Faculty of Medicine, “Victor Babeş” University of Medicine and Pharmacy, 300041 Timişoara, Romania; 4 Municipal Emergency Clinical Hospital of Timişoara, 300041 Timişoara, Romania

**Keywords:** Occipital artery, Computed tomography angiography, Atlanto-occipital interval, Far-lateral approach, Cerebrovascular bypass, Anatomical variation

## Abstract

**Purpose:**

The occipital artery (OA) is routinely encountered during posterior fossa and far-lateral craniocervical exposures and serves as a donor vessel for cerebrovascular bypass procedures. Despite its surgical importance, there is no standardised imaging-based classification of the OA course within the atlanto-occipital interval. This study aimed to characterise OA topography using computed tomography angiography (CTA) and develop a clinically applicable classification system.

**Methods:**

This retrospective observational study analysed 200 occipital arteries in 100 adult subjects using head-and-neck CTA. A dual-parameter classification was developed based on: (1) the primary type, describing the OA relationship to the occipital sulcus (Types 1–3), and (2) the vertical position relative to the atlas transverse process, including mastoid-transverse process overlap status.

**Results:**

Three primary types were identified based on the OA-sulcus relationship: Type 1 (intrasulcal), Type 2 (infrasulcal), and Type 3 (distanced). The vertical position was classified as supraatlantal, atlantal, or infraatlantal. Type 1 (intrasulcal) was most prevalent (54.0%), followed by Type 3 (distanced, 25.5%) and Type 2 (infrasulcal, 20.5%). Supraatlantal positioning predominated (61.5%). Type 1 arteries demonstrated significantly lower infraatlantal rates (5.6%) compared to Types 2 (26.8%) and 3 (25.5%) (*p* < 0.0001). Mastoid-transverse process overlap occurred in 15.5% and was independent of primary type. Bilateral type symmetry was present in 61.0% of subjects. Subset analysis (*n* = 30 subjects) revealed a mean OA luminal diameter of 1.29 ± 0.39 mm, with 88.3% of arteries demonstrating tortuosity.

**Conclusion:**

This CTA-based classification provides a structured framework for preoperative assessment of OA topography. The association between intrasulcal course and supraatlantal positioning may inform surgical planning for far-lateral exposures and OA harvest for bypass procedures.

## Introduction

The occipital artery (OA) is a vessel of paramount surgical significance within the suboccipital region and the craniocervical junction (CCJ). It functions as a critical structure requiring preservation during posterior fossa and far-lateral approaches, and it serves as a primary donor conduit for cerebrovascular bypass procedures [2, 4, 16]. Anatomically, the OA is divided into three distinct segments: the digastric segment, extending from its origin to the OA sulcus; the suboccipital (horizontal) segment, reaching the superior nuchal line (SNL); and the occipital (subgaleal) segment, located above the SNL [2]. Throughout its course, the artery maintains vital relationships with surrounding structures, including the posterior belly of the digastric muscle, the great vessels, the lower cranial nerves, and the greater occipital nerve (GON) [24].

While the OA typically originates from the posterior aspect of the external carotid artery (ECA) before entering the OA sulcus [1, 14], its trajectory is characterized by significant variability. Research indicates that in approximately 23% of cases, the OA arises above the gonial angle, and rarely, it may even originate from the internal carotid artery [11]. Recent computed tomography angiography (CTA) data further underscores this complexity, identifying 40 distinct branching sequences of the OA relative to other ECA branches [6]. Furthermore, aberrant trajectories, such as passage deep to the internal jugular vein or through the parotid gland, highlight the necessity of systematic preoperative imaging [21].

The surgical utility of the OA spans multiple disciplines, from neurosurgical OA-to-PICA bypasses, where vessel caliber and harvest length are decisive [18, 28], to regional anesthesia, where the artery serves as an ultrasound landmark for GON blocks [19]. Despite this, there remains a lack of standardized imaging classifications focusing on the OA’s course within the atlanto-occipital interval. Existing literature has largely prioritized segmental anatomy distal to the digastric muscle rather than the artery’s relationship to the mastoid-atlas complex [14, 24].

The present study addresses this clinical gap by proposing a dual-parameter classification system based on CTA. By characterizing the OA’s relationship to the OA sulcus, its vertical position relative to the atlas transverse process, and the presence of mastoid-atlas overlap, this classification provides a structured framework for surgical planning and modern harvest techniques.

## Materials and methods

### Study design and ethics

The present research followed the principles of the World Medical Association Code of Ethics (Declaration of Helsinki). The Ethical Committee (affiliation 4) approved this study (approval no. E-4247/15.10.2025). The study was conducted in accordance with current legislation, patient rights, and data protection regulations (GDPR Regulation no. 679/2016).

### Study population

Adult patients (≥ 18 years) who underwent head and neck CTA examinations with adequate visualisation of the bilateral occipital arteries within the atlanto-occipital interval were included. The sample comprised 100 consecutive subjects (200 occipital arteries) meeting these criteria.

### Inclusion and exclusion criteria

Subjects were excluded if they had: (1) prior surgical intervention in the suboccipital region; (2) congenital malformations of the craniovertebral junction; (3) traumatic injury affecting the occipital bone or upper cervical spine; (4) vascular pathology affecting the OA (occlusion, aneurysm, arteriovenous malformation); or (5) imaging artifacts precluding accurate assessment of the OA course.

### CTA protocol

CTA examinations were performed using a SOMATOM Definition Edge 128-slice multidetector CT scanner (Siemens Healthineers, Erlangen, Germany). The imaging protocol included: tube voltage 120 kVp, automatic tube current modulation (CARE Dose4D), slice thickness 0.625–1.0 mm, and pitch 0.5–1.0. Intravenous contrast agent (e.g., Iohexol 350 mgI/mL, 80–100 mL) was administered via power injector at 4.0–5.0 mL/s, with image acquisition triggered by bolus tracking in the aortic arch.

### Image analysis

All CTA datasets were reviewed using Horos v4.0.1 (The Horos Project, open-source DICOM viewer, https://horosproject.org) on a dedicated workstation. Multiplanar reconstructions (MPR) in axial, coronal, and sagittal planes, as well as maximum intensity projections (MIP) and volume rendering techniques, were employed to assess the OA’s course bilaterally.

Two experienced researchers (AMJ and MCR) independently evaluated each examination. Discrepancies were resolved by consensus review. The following anatomical landmarks were identified: (1) the OA sulcus; (2) the mastoid process tip; (3) the atlas (C1) transverse process.

### Classification system

**Fig. 1:**
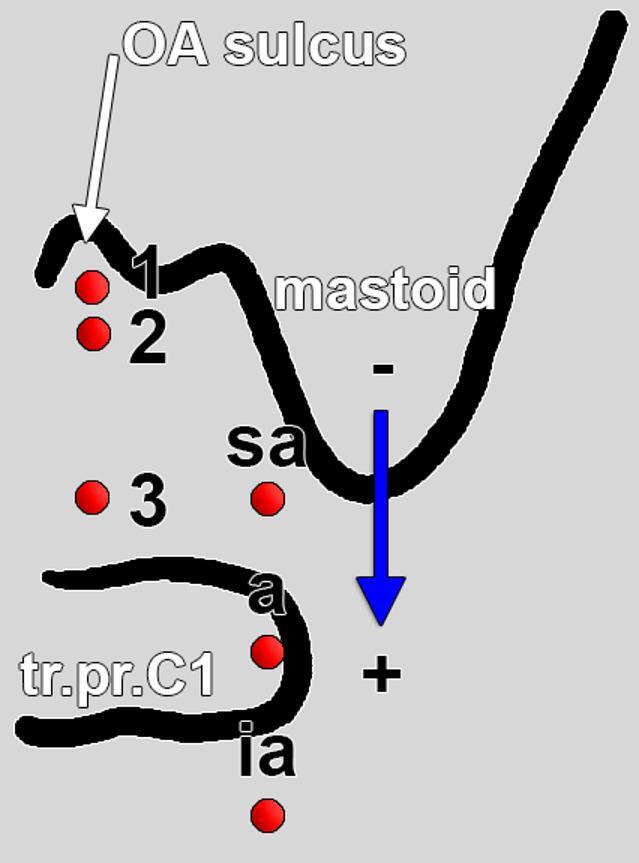
Diagram of the classification system of the occipital artery (OA) topographic variables in the atlantal-mastoid interval. Type 1: intrasulcal; Type 2: immediately infrasulcal; Type 3: distanced from the OA sulcus. The position of the OA referred to the transverse process of the atlas (tr.pr.C1) was classified: supraatlantal (sa), atlantal (a), and infraatlantal (i.a.). The overlap of the mastoid tip and the tr.pr.C1 was either negative (-) or positive (+)

A dual-parameter classification system was developed (Fig. 1) to characterise the OA course within the atlanto-occipital interval, based on: (1) the relationship to the OA sulcus (primary type), and (2) the vertical position relative to the atlas transverse process (subtype), including the presence or absence of mastoid-atlas overlap (Table 1).

The primary classification characterised the OA’s horizontal relationship to the OA sulcus. In Type 1 (intrasulcal), the OA runs within the sulcus itself, maintaining close apposition to the bony groove. In Type 2 (infrasulcal), the OA follows a course directly inferior to the sulcus, remaining in proximity but without lying within the groove. By contrast, Type 3 (distanced) denotes an OA trajectory that is distanced from the occipital sulcus, showing no direct groove-related alignment.

The subtype (mastoid-atlas topography) reflects the OA’s vertical relationship to the C1 transverse process at the mastoid level, incorporating mastoid-transverse process of C1 overlap (coronal overlap of the mastoid tip and C1 transverse process). For Types 1–2, four subtypes apply: a (supraatlantal) above C1; b (atlantal with overlap) at C1 where overlap is present; c (atlantal without overlap) at C1 where overlap is absent; and d (infraatlantal) below C1, with a (+/−) modifier indicating overlap present/absent. For Type 3, three subtypes apply: a (supraatlantal) above C1, b (atlantal) at C1, and c (infraatlantal) below C1, with overlap recorded as (+) present or (−) absent.

### Statistical analysis

Statistical analyses were performed using Python 3.x with SciPy (v1.x) and pandas (v2.x) libraries. Descriptive statistics included frequencies and percentages for categorical variables. Chi-square (χ²) tests were used to assess associations between categorical variables, including: (1) right versus left side type distribution; (2) primary type versus vertical position; (3) primary type versus overlap status; and (4) sex versus type distribution. Fisher’s exact test was applied when expected cell frequencies were < 5. Bilateral symmetry was assessed using McNemar’s test to evaluate whether there was a systematic side preference for specific classifications. Cohen’s kappa (κ) coefficient was calculated to quantify the degree of bilateral agreement, interpreted as: <0.20 poor, 0.21–0.40 fair, 0.41–0.60 moderate, 0.61–0.80 substantial, and > 0.80 almost perfect agreement. A two-tailed p-value < 0.05 was considered statistically significant. All confidence intervals were calculated at the 95% level.

### Subset analysis: luminal diameter and tortuosity

In a randomly selected subset of 30 subjects (60 arteries), additional morphometric assessments were performed to characterise OA calibre and tortuosity. Luminal diameter was measured at the mid-height of the mastoid process using electronic calipers on axial CTA images. Tortuosity was qualitatively assessed and classified as: straight (no deviation from a direct course), mildly tortuous (gentle curves without acute angulation), markedly tortuous (prominent curves with angulation), kink (acute angular bend), or coil (complete loop or spiral configuration).

**Table 1:** Summary of the OA classification system

Type	Subtype	Vertical position	Overlap status
1	a	Supraatlantal	N/A (above atlas)
	b	Atlantal	WITH mastoid-TP overlap
	c	Atlantal	WITHOUT mastoid-TP overlap
	d+/-	Infraatlantal	+ with overlap / - without
2	a	Supraatlantal	N/A (above atlas)
	b	Atlantal	WITH mastoid-TP overlap
	c	Atlantal	WITHOUT mastoid-TP overlap
	d+/-	Infraatlantal	+ with overlap / - without
3	a+/-	Supraatlantal	+ with overlap / - without
	b+/-	Atlantal	+ with overlap / - without
	c+/-	Infraatlantal	+ with overlap / - without

TP = transverse process; N/A = not applicable. Note: For Types 1 and 2, subtypes b and c encode overlap status directly; for Type 3, overlap is indicated by +/- modifier

## Results

### Demographics

The study population comprised 100 adult subjects (200 occipital arteries), including 55 males (55.0%) and 45 females (45.0%). All subjects met the inclusion criteria and provided adequate bilateral CTA imaging for anatomical assessment.

### Interobserver agreement

Two experienced researchers independently classified all CTA examinations. Initial ratings were fully concordant for all categorical endpoints (primary type, vertical position, mastoid–transverse process overlap status, and the composite classification code), resulting in 100% observed agreement and Cohen’s κ = 1.000 at each level. Accordingly, the consensus review served only as confirmation and no third-party adjudication was required.

### Primary type distribution

Analysis of the OA-to-sulcus relationship revealed three distinct primary types across the pooled sample of 200 arteries. Type 1 (intrasulcal course) was the most prevalent, observed in 108 arteries (54.0%), followed by Type 3 (course distanced from the sulcus) in 51 arteries (25.5%), and Type 2 (immediately infrasulcal course) in 41 arteries (20.5%).

The distribution of primary types showed remarkable consistency between sides. On the right side (*n* = 100), Type 1 was identified in 54 arteries (54.0%), Type 2 in 20 arteries (20.0%), and Type 3 in 26 arteries (26.0%). The left side (*n* = 100) showed a nearly identical pattern: Type 1 in 54 arteries (54.0%), Type 2 in 21 arteries (21.0%), and Type 3 in 25 arteries (25.0%). Chi-square analysis confirmed no statistically significant difference between right and left side distributions (χ² = 0.044, df = 2, *p* = 0.978).

### Vertical position distribution (mastoid-atlas topography)

The mastoid-atlas topographic assessment classified occipital arteries according to their vertical position relative to the atlas transverse process. The subtypes differ by primary type: for Types 1 and 2, subtypes a (supraatlantal), b (atlantal with mastoid-transverse process overlap), c (atlantal without overlap), and d (infraatlantal) are defined; for Type 3, subtypes a (supraatlantal), b (atlantal), and c (infraatlantal) are used.

Pooled analysis (*n* = 200) revealed supraatlantal positioning as the most common, present in 123 arteries (61.5%). Atlantal-level positioning was observed in 47 arteries (23.5%), while infraatlantal positioning (subtypes 1d, 2d, or 3c) was present in 30 arteries (15.0%).

Side-specific analysis demonstrated similar patterns. On the right side, 58 arteries (58.0%) were supraatlantal, 26 (26.0%) atlantal, and 16 (16.0%) infraatlantal. The left side showed 65 arteries (65.0%) supraatlantal, 21 (21.0%) atlantal, and 14 (14.0%) infraatlantal.

### Type × vertical position association

Cross-tabulation analysis revealed a highly significant association between primary type and vertical position (χ² = 33.574, df = 4, *p* < 0.0001). Each primary type demonstrated a distinctive vertical position profile.

Type 1 arteries (*n* = 108) showed predominantly supraatlantal positioning: 82 (75.9%) supraatlantal, 20 (18.5%) atlantal, and 6 (5.6%) infraatlantal. Type 2 arteries (*n* = 41) demonstrated the highest rate of infraatlantal positioning: 11 (26.8%), 24 (58.5%), and 6 (14.6%) were supraatlantal, atlantal, and infraatlantal, respectively. Type 3 arteries (*n* = 51) showed an intermediate pattern with 17 (33.3%) supraatlantal, 21 (41.2%) atlantal, and 13 (25.5%) infraatlantal.

A key finding was that Type 1 (intrasulcal) arteries have a significantly lower infraatlantal rate (5.6%) compared to Type 2 (26.8%) and Type 3 (25.5%). This suggests that the intrasulcal course is associated with a more cranial (supraatlantal) trajectory through the atlanto-occipital interval.

### Mastoid-transverse process of C1 overlap status

Overlap between the mastoid process tip and the atlas transverse process was uncommon, being absent in 169/200 arteries (84.5%) and present in 31/200 (15.5%; 95% CI, 11.1–21.2). Overlap was observed more frequently on the right (19/100, 19.0%; 95% CI, 12.5–27.8) than on the left (12/100, 12.0%; 95% CI, 7.0–19.8).

At the subject level (*n* = 100), overlap was bilateral in 8 subjects (8.0%), unilateral in 15 (15.0%), and absent bilaterally in 77 (77.0%). Among unilateral cases, right-sided overlap predominated (11 vs. 4 subjects). Paired comparison using the exact McNemar test showed no significant laterality for overlap (b = 11, c = 4; *p* = 0.237). Overall interside concordance for overlap status was 85.0%, corresponding to moderate agreement (Cohen’s κ = 0.433).

When stratified by primary type (*n* = 200 arteries), overlap rates were 14.8% for Type 1 (16/108), 22.0% for Type 2 (9/41), and 11.8% for Type 3 (6/51). This distribution did not differ significantly across types (χ² = 1.885, df = 2, *p* = 0.390).

### Bilateral symmetry analysis

Bilateral symmetry was assessed at multiple classification levels. Complete classification symmetry (identical codes on both sides) was observed in 42 subjects (42.0%), while 58 subjects (58.0%) demonstrated asymmetric patterns.

At the primary type level, bilateral symmetry increased to 61 subjects (61.0%), with the most common symmetric patterns being Type 1/1 (38 subjects, 38.0%), Type 3/3 (14 subjects, 14.0%), and Type 2/2 (9 subjects, 9.0%). Among asymmetric combinations (39 subjects, 39.0%), the most frequent were Type 1/2 and Type 2/1 (8 subjects each, 8.0%), and Type 1/3 and Type 3/1 (8 subjects each, 8.0%).

Vertical position symmetry (same vertical level bilaterally) was present in 61 subjects (61.0%), while overlap status concordance was highest at 85 subjects (85.0%).

McNemar’s test for Type 1 versus non-Type 1 classification showed no systematic side preference (discordant pairs: b = 16, c = 16; *p* = 1.000). Cohen’s kappa coefficients indicated fair bilateral concordance between right and left sides for the full classification (κ = 0.270), primary type (κ = 0.352), and vertical position (κ = 0.286), with moderate concordance for overlap status (κ = 0.433).

### Sex analysis

Comparison of primary type distribution between males (*n* = 55 subjects, 110 arteries) and females (*n* = 45 subjects, 90 arteries) revealed no statistically significant difference (χ² = 1.142, df = 2, *p* = 0.565). In males, Type 1 was observed in 56 arteries (50.9%), Type 2 in 23 (20.9%), and Type 3 in 31 (28.2%). Females showed Type 1 in 52 arteries (57.8%), Type 2 in 18 (20.0%), and Type 3 in 20 (22.2%).

### Infraatlantal course analysis

Infraatlantal course (subtype d for Types 1 and 2, or subtype c for Type 3) was observed in 30 arteries (15.0%). The distribution varied significantly by primary type: Type 1 arteries showed the lowest infraatlantal rate at 6/108 (5.6%), while Types 2 and 3 demonstrated substantially higher rates at 11/41 (26.8%) and 13/51 (25.5%), respectively.

**Fig. 2:**
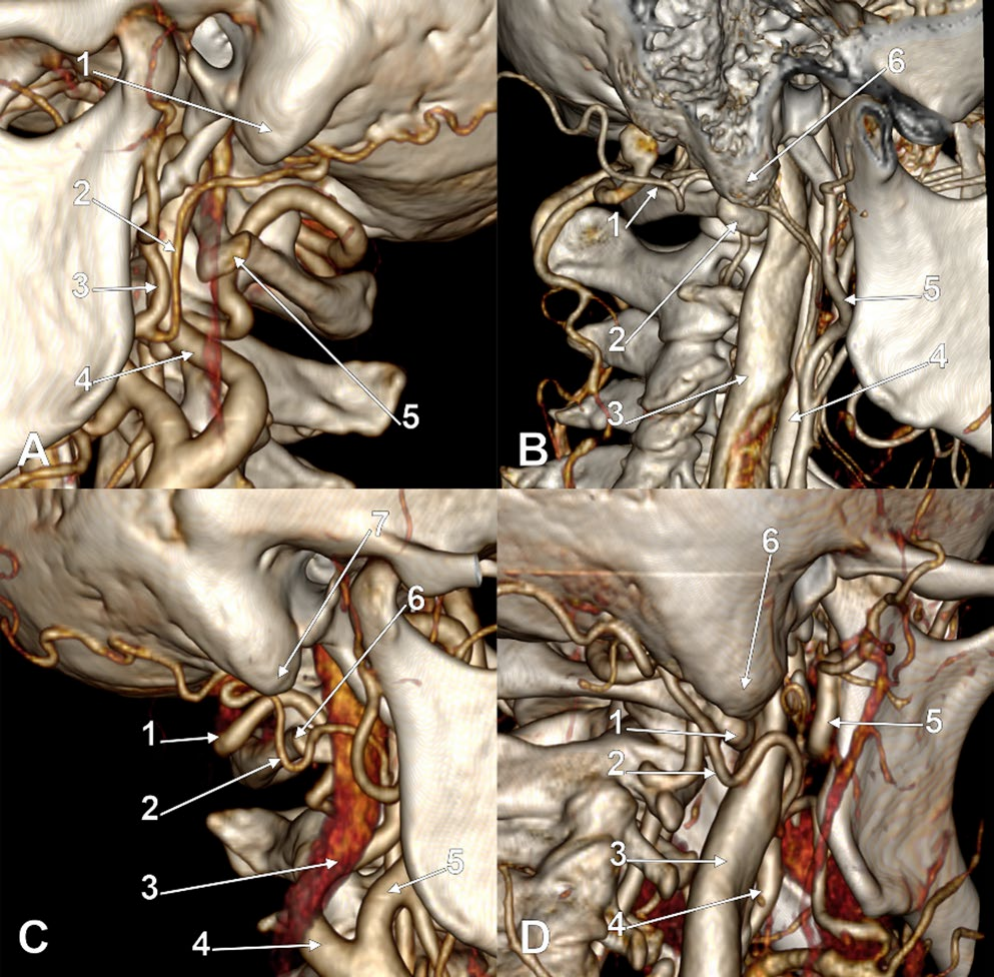
A. Left occipital artery of Type 1a. Three-dimensional volume rendering. Infero-lateral view. (1) mastoid tip; (2) occipital artery; (3) external carotid artery; (4) internal carotid artery; (5) transverse process of the atlas. B. Right occipital artery of Type 1b. Three-dimensional volume rendering. Postero-infero-lateral view. (1) occipital artery; (2) transverse process of the atlas; (3) internal jugular vein; (4) internal carotid artery; (5) external carotid artery; (6) mastoid tip. C. Right occipital artery of Type 1c. Three-dimensional volume rendering. Infero-lateral view. (1) vertebral artery; (2) occipital artery; (3) internal jugular vein; (4) internal carotid artery; (5) external carotid artery; (6) transverse process of the atlas; (7) mastoid tip. D. Right occipital artery of Type 1d+. Three-dimensional volume rendering. Postero-infero-lateral view. (1) transverse process of the atlas; (2) occipital artery; (3) internal jugular vein; (4) internal carotid artery; (5) external carotid artery; (6) mastoid tip

**Fig. 3:**
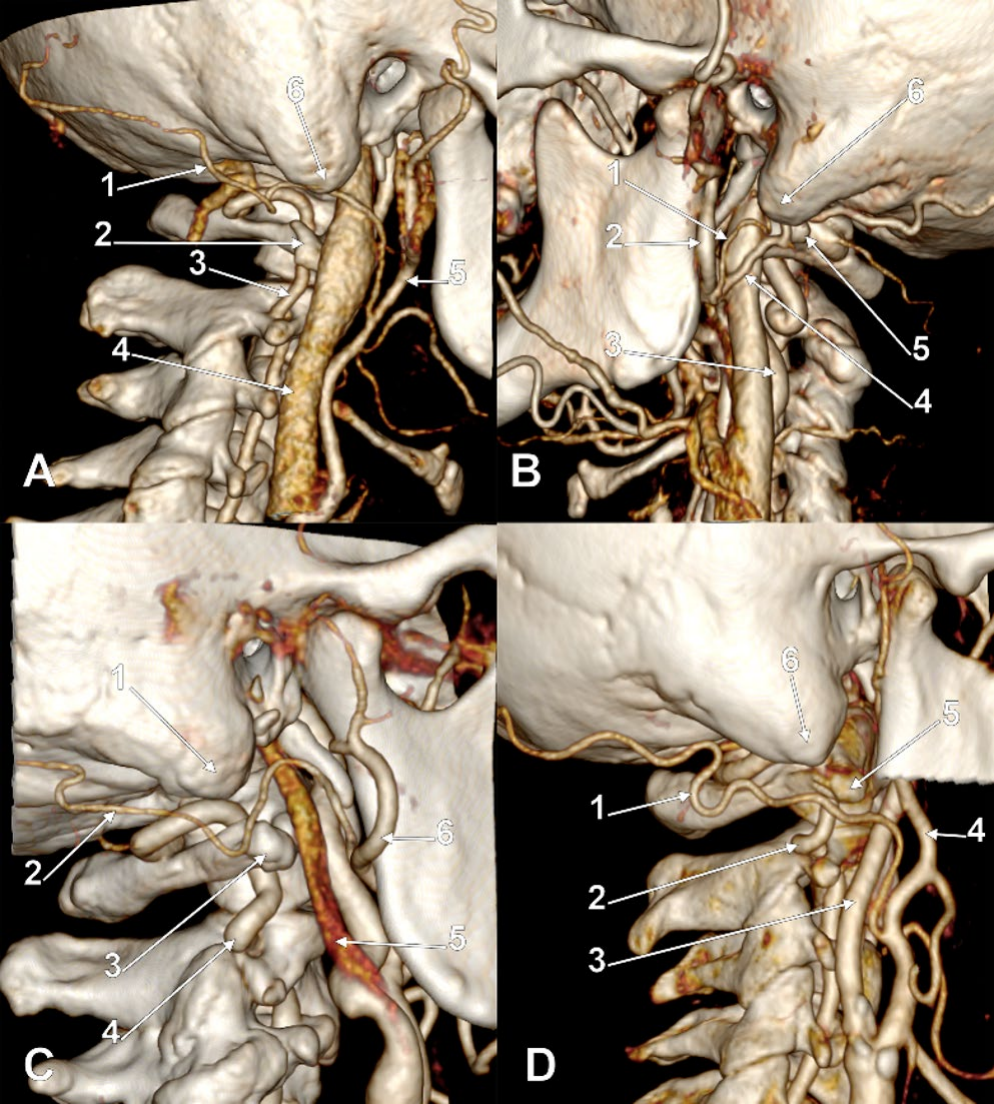
A. Right occipital artery of Type 2a. Three-dimensional volume rendering. Postero-infero-lateral view. (1) occipital artery; (2) transverse process of the atlas; (3) vertebral artery; (4) internal jugular vein; (5) external carotid artery; (6) mastoid tip. B. Left occipital artery of Type 2b. Three-dimensional volume rendering. Infero-lateral view. (1) posterior auricular artery; (2) external carotid artery; (3) internal carotid artery; (4) occipital artery; (5) transverse process of the atlas; (6) mastoid tip. C. Right occipital artery of Type 2c. Three-dimensional volume rendering. Postero-infero-lateral view. (1) mastoid tip; (2) occipital artery; (3) transverse process of the atlas; (4) vertebral artery; (5) internal jugular vein; (6) external carotid artery. D. Right occipital artery of Type 2d+. Three-dimensional volume rendering. Postero-infero-lateral view. (1) occipital artery; (2) vertebral artery; (3) internal carotid artery; (4) external carotid artery; (5) transverse process of the atlas; (6) mastoid tip

**Fig. 4:**
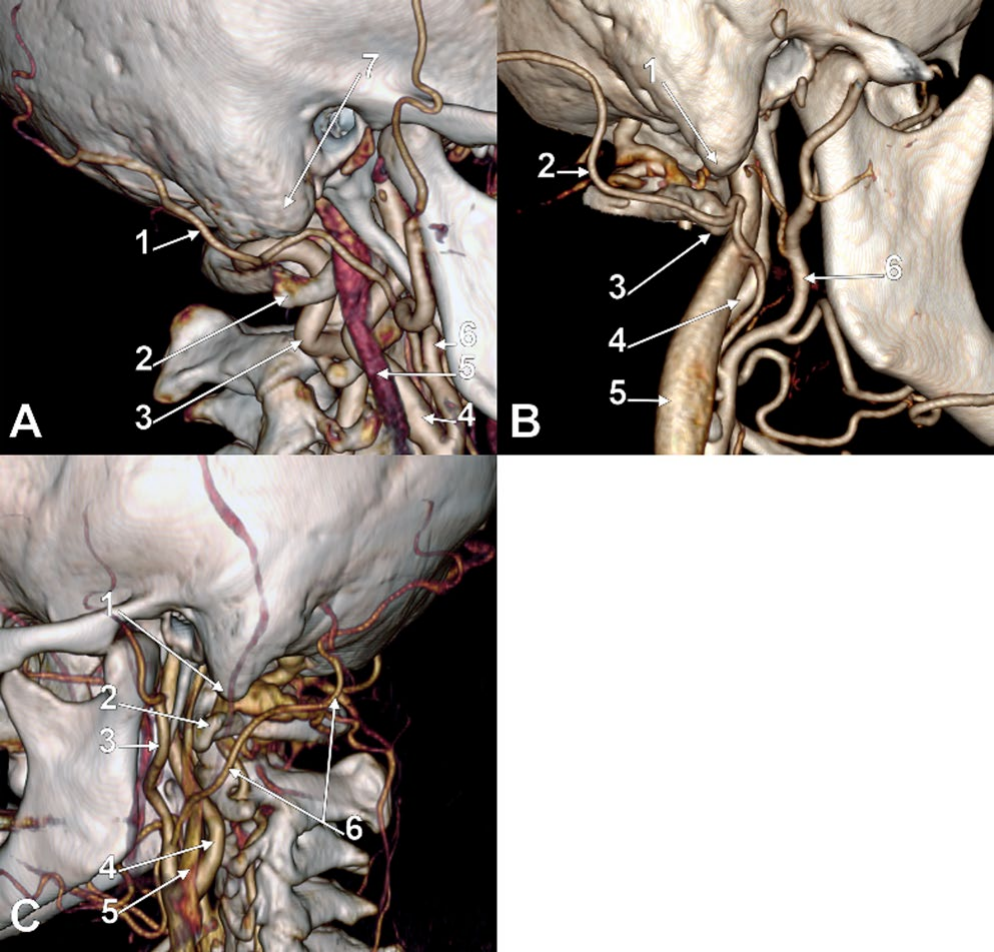
A. Right occipital artery of Type 3a-. Three-dimensional volume rendering. Postero-infero-lateral view. (1) occipital artery; (2) transverse process of the atlas; (3) vertebral artery; (4) internal carotid artery; (5) internal jugular vein; (6) external carotid artery; (7) mastoid tip. B. Right occipital artery of Type 3b-. Three-dimensional volume rendering. Postero-infero-lateral view. (1) mastoid tip; (2) occipital artery; (3) transverse process of the atlas; (4) internal carotid artery; (5) internal jugular vein; (6) external carotid artery. C. Left occipital artery of Type 3c-. Three-dimensional volume rendering. Postero-infero-lateral view. (1) mastoid tip; (2) transverse process of the atlas; (3) external carotid artery; (4) internal carotid artery; (5) internal jugular vein; (6) occipital artery

### Complete classification frequencies

The combined type-subtype classification yielded 17 distinct anatomical variants. The most prevalent was 1a (intrasulcal, supraatlantal; Fig. 2A) at 82 arteries (41.0%), followed by 2a (immediately infrasulcal, supraatlantal; Fig. 3A) at 24 (12.0%), 3b- (distanced, atlantal without overlap; Fig. 4B) at 18 (9.0%), 3a- (distanced, supraatlantal without overlap; Fig. 4A) at 15 (7.5%), 1b (intrasulcal, atlantal with overlap; Fig. 2B) at 14 (7.0%), and 3c- (distanced, infraatlantal without overlap; Fig. 4C) at 11 (5.5%). Less common variants included 1c (intrasulcal, atlantal without overlap; Fig. 2C), 2b (immediately infrasulcal, atlantal with overlap; Fig. 3B), 2c (immediately infrasulcal, atlantal without overlap; Fig. 3C), 1d+ (intrasulcal, infraatlantal with overlap; Fig. 2D), and 2d+ (immediately infrasulcal, infraatlantal with overlap; Fig. 3D). The remaining variants each accounted for less than 5% of the sample.

### Subset analysis: luminal diameter and tortuosity

#### Luminal diameter

In a randomly selected subset of 30 subjects (60 arteries), luminal diameter was measured at mid-mastoid level. Overall mean luminal diameter was 1.29 ± 0.39 mm (range 0.49–2.95 mm). Right side diameter (1.27 ± 0.31 mm) did not differ significantly from left side (1.30 ± 0.47 mm; paired t-test, *p* = 0.675).

Luminal diameter by primary type was: Type 1, 1.36 ± 0.46 mm (*n* = 31); Type 2, 1.24 ± 0.20 mm (*n* = 14); Type 3, 1.17 ± 0.36 mm (*n* = 15). No significant difference was observed between types (ANOVA, F = 1.309, *p* = 0.278).

#### Tortuosity

In the same subset, tortuosity was qualitatively assessed. The distribution was: straight, 7 (11.7%); mildly tortuous, 20 (33.3%); markedly tortuous, 21 (35.0%); kink, 3 (5.0%); coil, 9 (15.0%). Overall, 53/60 arteries (88.3%) demonstrated some degree of tortuosity. Severe tortuosity (kink or coil) was present in 12/60 arteries (20.0%).

When stratified by primary type, Type 1 arteries showed the highest rate of straight morphology: Type 1, 7/31 straight (22.6%); Type 2, 0/14 straight (0%); Type 3, 0/15 straight (0%). This association between primary type and tortuosity was statistically significant (χ² = 17.08, df = 8, *p* = 0.029) (Table 2).

**Table 2:** Luminal diameter and tortuosity in a 30-subject subset (60 arteries)

Parameter	Value
*Luminal diameter (mm)*	
Overall	1.29 ± 0.39 (range 0.49–2.95)
Right side	1.27 ± 0.31
Left side	1.30 ± 0.47
Type 1 (*n* = 31)	1.36 ± 0.46
Type 2 (*n* = 14)	1.24 ± 0.20
Type 3 (*n* = 15)	1.17 ± 0.36
*Tortuosity, n (%)*	
Straight	7 (11.7%)
Mildly tortuous	20 (33.3%)
Markedly tortuous	21 (35.0%)
Kink	3 (5.0%)
Coil	9 (15.0%)
*Summary*	
Any tortuosity	53/60 (88.3%)
Severe tortuosity (kink/coil)	12/60 (20.0%)

## Discussion

This study provides a CTA-based characterisation of the OA at the mastoid and atlanto-occipital interval, with particular attention to the digastric segment and its relationship to the OA sulcus, the mastoid tip, and the transverse process of the atlas (Table 3). Because the OA is routinely encountered during posterior and far-lateral craniocervical exposures and may also serve as a donor or recipient in revascularisation procedures, imaging variables that predict its topography are directly relevant to surgical planning and intraoperative risk mitigation [2, 4, 16, 22, 25].

**Table 3:** Summary of key findings. TP = transverse process of atlas

Parameter	*n*	%
*Study population*		
Total subjects	100	–
Total arteries	200	–
*Primary Type (OA-sulcus relationship)*		
Type 1 (intrasulcal)	108	54.0
Type 2 (infrasulcal)	41	20.5
Type 3 (distanced)	51	25.5
*Vertical Position*		
Supraatlantal	123	61.5
Atlantal	47	23.5
Infraatlantal	30	15.0
*Other Parameters*		
Bilateral type symmetry	61	61.0
Mastoid-TP overlap	31	15.5

### Surgical relevance of the sulcus-based primary type (OA–occipital sulcus relationship)

The OA sulcus, situated on the occipitomastoid suture (OMS), serves as a vital topographic landmark for identifying the OA during suboccipital dissections [1]. Before reaching this sulcus, the OA traverses the condylar triangle, a space defined by the rectus capitis lateralis (anteriorly), the superior oblique (posteriorly), and the occipital bone (superiorly). As noted by Cohen et al. (2017), this triangle provides a surgical corridor to the vertebral artery and occipital condyle that bypasses the traditional suboccipital triangle [8]. Within this region, the OA typically runs between the rectus capitis lateralis and the posterior digastric belly. While this serves as a reliable guide for exposure or ligation, our findings indicate significant topographical variability of the OA within the condylar triangle.

It should be noted that the occipital sulcus itself demonstrates morphological variability. Alvernia et al. (2006) reported that the OA occupies a true bony canal in 66% of specimens versus an open groove in 34%, indicating that sulcus depth and configuration are not constant. In some individuals, the sulcus may be shallow or poorly defined. Our classification accounts for this variability: Type 1 indicates an intrasulcal course when a well-formed sulcus is present, while Types 2 and 3 describe OA positions that do not depend on sulcus presence or definition for their identification.

The OA sulcus-based primary type in the present study describes how closely the OA follows its bony groove, a landmark frequently used to locate and mobilise the vessel during exposure and harvest. The OMS was found medial to the OAS 68.6% of cases [4]. That study was performed on sixteen cadavers and the authors did not find or report the variable distance between the OA and its sulcus, such as we found here [4].

Techniques that emphasise bony and periosteal cues, such as transperiosteal “inside-out” strategies, orientational harvest, and intermuscular harvest along far-lateral exposures, rely on predictable OA relationships to the bony sulcus, digastric notch, and adjacent muscular envelopes [2, 4, 16, 22, 25]. Within this surgical logic, an intrasulcal course (Type 1) enables rapid localisation using the sulcus itself. In contrast, an immediately infrasulcal or a distanced course (Types 2–3) implies that the artery may be encountered slightly off the groove axis and therefore requires a broader soft-tissue survey to avoid missed identification or inadvertent injury during dissection or drilling.

### Catheter placement via the occipital artery: implications of OA topography

Beyond its established role in posterior fossa and bypass-oriented surgery, the OA has also been used as a conduit for continuous intra-arterial infusion when catheterisation via the superficial temporal artery is not feasible. In a technical note, Hasegawa et al. described a posterior mastoid approach for OA exposure and catheter placement, supported by routine preoperative CTA, reporting successful catheter placement in all 15 patients without major wound complications [15].

Several elements of that technique map directly onto the present CTA-based topographic framework. The mastoid process is used as the dominant bony reference to reach the OA in a deep muscular plane, where adjacent venous anatomy may complicate haemostasis [15]. In our cohort, Type 1 (intrasulcal) arteries accounted for 54.0% and exhibited a distinctly cranial vertical profile (infraatlantal 5.6% vs. 26.8% and 25.5% for Types 2 and 3; *p* < 0.0001), supporting the expectation that a groove-guided, mastoid-referenced search will be efficient in the majority of cases. Conversely, the 15.0% overall prevalence of infraatlantal courses and the groove-distanced configurations highlight a substantive subset in which the OA may be encountered lower and/or further from the anticipated sulcus axis, strengthening the rationale for preoperative CTA planning when the OA is selected for catheter access [15].

A mastoid tip–atlas transverse process overlap occurred in 15.5% and was independent of primary type, suggesting that corridor crowding can modify exposure even when the OA follows a favourable sulcus-based pattern. It should be considered when planning retraction, haemostasis, and catheter securitisation around the mastoid-C1 junction.

### Segmental anatomy and OA-PICA bypass considerations

The OA distal to the posterior belly of the digastric muscle traverses distinct tissue planes that must be recognised during harvest. The classic three-segment model proposed by Alvernia et al. (2006), comprising the digastric, suboccipital (horizontal), and occipital (subgaleal) segments, provides a useful framework [2]. In the digastric segment, the OA diameter at origin ranged from 2.2 to 2.93 mm; in 66% of specimens, the artery ran in a true bony canal, which we did not find, while in 34% it occupied a groove [2]. However, these authors did not present evidence supporting that canal of the OA, defined the branches of the OA for the facial, accessory and hypoglossal nerves as „neuronal branches”, and did not observe or report a variable vertical topography of the digastric segemt of the OA [2]. At the exit from the OA sulcus, the mean diameter of the artery was 1.9 ± 0.2 mm, decreasing to 1.4 ± 0.3 mm at the SNL [2]. Critically, the OA maintained a diameter greater than 1.0 mm for an average distance of 50 ± 12 mm above the SNL, providing sufficient calibre for bypass anastomosis [2]. Nossek et al. (2014) describe a refined three-segment model for the distal OA: an intramuscular segment running between the splenius capitis and semispinalis capitis muscles, a transitional segment crossing multiple vertically superimposed tissue layers, including the sternocleidomastoid tendon and galea aponeurotica, and a subcutaneous segment above the SNL [18]. Dissection of the OA is technically challenging, particularly in the transitional segment, and requires microscopic dissection with repeated microdoppler evaluations to avoid injury to vessels [18]. It was reported a mean OA diameter of 1.65 mm at the SNL, with an available harvest length of 8.24 cm, dimensions suitable for posterior circulation revascularisation [28].

The OA-to-PICA bypass is among the more demanding cerebrovascular procedures. It was recommended dissecting 10–12 cm of OA to reach the anastomosis site without tension [18]. It was shown that the OA-p1 PICA end-to-end bypass offers favourable calibre matching (OA 1.65 mm vs. p1 segment 1.74 mm) and requires a shorter direct bypass distance (3.12 cm) than the OA-p3 PICA end-to-side approach (4.89 cm) [28]. PICA’s p1 segment carries fewer perforators (median 1) than the p3 segment (median 4), potentially reducing the risk of ischaemic brainstem injury during manipulation [28].

### Vertical position relative to the atlas: implications for craniocervical exposure and OA mobilisation

Far-lateral and anterolateral routes to the craniocervical junction are organised around the compact mastoid–occipital–atlas complex. In these approaches, the C1 transverse process is a key intraoperative landmark situated just inferior and anterior to the mastoid tip, and the V3 segment of the vertebral artery is exposed by dividing the small suboccipital muscular insertions around C1 and the posterior arch [5, 20]. Microanatomical analyses of far-lateral corridors further demonstrate how condylar, paracondylar and mastoid-related bony constraints shape the working angles and the vascular relationships in this region [9, 10, 13, 20].

Against this background, the vertical position variable in our classification provides a practical descriptor of where the OA travels in relation to the C1 transverse process and, by extension, where it is likely to intersect the surgical field during suboccipital dissection. A supraatlantal trajectory implies that the vessel remains more cranial within the mastoid-occipital envelope. In contrast, atlantal-level and infraatlantal trajectories imply progressively more caudal passage through the atlanto-occipital interval, potentially increasing the likelihood of early OA encounter during deeper muscle splitting and exposure of C1-related landmarks. These positional differences are therefore relevant both for protecting the OA during far-lateral exposure and for planning its mobilisation when an OA conduit is required [2, 16, 22].

### Mastoid tip–atlas transverse process overlap: a surrogate of corridor tightness at the craniocervical junction

The mastoid tip–C1 transverse process relationship is a recognised determinant of the available bony corridor and soft-tissue working triangle at the craniocervical junction. When the mastoid tip projects over the transverse process of the atlas, the operative space around the V3 segment can become more constrained, with tighter packing between mastoid, atlas, and occipital condyle during far-lateral exposure and drilling [5, 20]. Contemporary refinements in exposure techniques for the horizontal part of V3 further underscore the importance of understanding patient-specific soft-tissue and bony constraints in this region [17].

In our cohort, the mastoid-transverse process of C1 overlap was present in a minority of cases . It showed no statistically significant association with the primary type of OA, supporting the interpretation that overlap represents an independent, corridor-level modifier rather than a simple determinant of OA-to-sulcus relationship. Clinically, this modifier should be regarded as a warning feature for potentially reduced manoeuvring space during deep dissection around C1 and the mastoid tip, where the OA and VA-related corridors converge. Of particular note, anastomotic channels between the OA and the vertebral artery through radicular branches are present in approximately 91% of individuals, typically involving the superficial or deep descending branches of the OA and the posterior radicular arteries at C1, C2, or C3 levels [2]. These anastomoses become clinically relevant during therapeutic embolisation of lesions fed by the OA, where inadvertent embolisation through these channels can result in posterior circulation stroke. Given that osseous and vertebral artery anomalies at the craniovertebral junction are readily appreciable on CT/CTA, systematic preoperative assessment of these relationships is advisable when planning far-lateral trajectories or vascular procedures involving the OA or V3 [2, 12, 23, 27].

### Bilaterality and side-to-side predictability: implications for preoperative mapping

Although side-specific distributions of the primary types were broadly similar, complete code-level symmetry was not universal. This limits the reliability of using the contralateral side as a surrogate for planning dissection or harvest on the side of interest. From an operative standpoint, the practical consequence is that side-specific preoperative mapping of the OA course and its relationship to the mastoid tip and atlas transverse process should be preferred, particularly when the OA is planned for mobilisation, bypass preparation, or preservation during complex far-lateral exposure [23, 27].

### Practical synthesis for surgical planning

Taken together, the variables reported in this study offer a structured CTA framework that aligns with established operative landmark strategies. Specifically, combining (i) the OA-OA sulcus relationship, (ii) the vertical position relative to the atlas transverse process, and (iii) the presence or absence of mastoid-C1 overlap can inform expectations regarding where the OA will be encountered during muscle splitting, how readily it may be traced using groove-based landmarks, and whether the deep corridor is likely to be crowded during exposure around C1 and the V3 segment. In settings where an OA conduit is required, these variables also support a more deliberate selection of the side and the dissection plane along the mastoid–occipital envelope, consistent with microanatomical descriptions of OA segmentation and harvest strategies [2, 4, 16, 22, 25].

### Implications for regional anaesthesia and neurovascular relationships in the occiput

The OA also plays an important role in scalp block procedures for posterior cranial surgery. Ultrasound-guided blockade of the GON can be achieved by visualising the OA and injecting local anaesthetic adjacent to it, as the nerve consistently accompanies the artery along its course [19]. This technique provides dense, prolonged analgesia for suboccipital procedures.

Recent anatomical studies have refined our understanding of the GON-OA relationship in the occiput. Won et al. (2018) examined 56 halved heads. They found that the GON and OA pierced the fascia within a 2 cm radius circle centered on the medial trisection point of the external occipital protuberance-mastoid process line in 85.7% and 98.2% of specimens, respectively [26]. Critically, the GON and OA were in the same sector of this circle in only 51.8% of cases, whereas in 33.9% they pierced different sectors [26]. The OA pierced the fascia at 17.0 ± 9.2 mm below the external occipital protuberance and 33.7 ± 9.9 mm lateral to the midsagittal line, and was typically lateral to the GON in 85.7% of specimens [26]. These findings indicate that the OA cannot be relied upon as a universal landmark for GON blockade in all patients. False-positive or false-negative responses may occur even with ultrasound guidance.

The relationship between the OA and adjacent neural structures is also clinically relevant in microvascular decompression surgery for trigeminal neuralgia. Chen et al. (2018) measured the distance from the OA to the projection of the transverse sinus at 3.2 ± 0.6 cm at the mastoid groove and 2.5 ± 0.4 cm at a site 0.5–1.0 cm medial to the mastoid groove. They demonstrated that making skin incisions above the OA, detected preoperatively with Doppler ultrasound, significantly reduced postoperative sensory disturbance by protecting the lesser occipital nerve (0% vs. 44% at 3-month follow-up, *p* = 0.002) [7]. The OA thus serves as a practical landmark for determining the inferior extent of retrosigmoid incisions while simultaneously protecting adjacent cutaneous nerves.

Knowledge of the OA course variability, as characterised in the present study for the digastric segment, may assist in optimising needle placement during regional anaesthesia and in planning surgical incisions that avoid both vascular and neural complications. The relationship between the GON and the trapezius muscle adds further complexity. It was found that the GON passed the lateral border of the trapezius muscle before piercing the overlying fascia in 62.5% of cases, whereas in 37.5% it pierced the muscle itself, a potential entrapment site relevant to cervicogenic headache [26].

### Variability of OA origin and aberrant courses: implications for proximal identification

The present classification focuses on the OA within the atlanto-occipital interval; however, surgeons should also be aware of proximal variability. The vertical level of OA origin from the ECA is not constant. It was demonstrated that the OA origin ranges from infrahyoid (1.1%) to supragonial levels (23.3%), with bilateral symmetry in only 56.7% of subjects [11]. The most common origin level is infragonial (40.6%), followed by gonial (28.3%) [11]. In rare cases (1.1%), the OA may originate from the internal carotid artery, a variant that must be recognised to avoid inadvertent injury during carotid interventions [11].

Beyond vertical origin variability, the sequential position of the OA within the ECA branching pattern also demonstrates remarkable diversity. Calotă et al. (2025) classified ECA branching into 40 distinct sequential types (S-types) in 170 carotid axes, revealing that the OA may arise at various positions relative to the STA, LA, FA, and APA. In the most common patterns, the OA originated as the second branch (type 1: STA-OA-LA-APA-FA, 1.2%), as the fourth branch (type 4: STA-LA-APA-OA-FA, 5.3%; type 7: STA-LFT-APA-OA, 7.1%; type 8: STA-LA-APA-FA-OA, 6.5%), or as part of an occipitopharyngeal trunk with the ascending pharyngeal artery (OPT, 17.06% of sides). Importantly, the OPT, when present, appeared within seven distinct S-type patterns, most commonly as type 9 (STA-LA-OPT-FA, 6.5%) and type 10 (STA-OPT-LA-FA, 5.9%). No statistically significant side-related or gender-related differences were found in S-type distribution (*p* = 0.379 and *p* = 0.138, respectively), indicating that surgeons cannot predict contralateral OA branching patterns from ipsilateral findings [6].

Furthermore, aberrant courses of the OA have recently been documented, including passage deep to the internal jugular vein and retromandibular trajectories through the parotid gland [21]. An intraparotid OA represents a plausible site for vascular pathology and should be considered in the differential diagnosis of parotid swellings. These variants emphasise that the OA lacks a single reliable landmark for identification, and that a comprehensive preoperative CTA assessment extending from the carotid bifurcation to the suboccipital region is advisable when the OA is relevant to the planned procedure.

### The type 1 profile: a potentially favourable configuration

The finding that Type 1 (intrasulcal) arteries have significantly lower infraatlantal rates (5.6%) compared to Types 2 and 3 (approximately 26% each) has practical implications. An intrasulcal OA is more predictable in location, being reliably identified using the OA sulcus as a bony landmark. Additionally, such arteries are less likely to be encountered during deep dissection around the C1 transverse process. This combination may represent a “favourable” anatomical profile for both protective strategies during far-lateral exposure and for harvest planning when an OA conduit is required. Conversely, Types 2 and 3 arteries require a broader soft-tissue survey for identification and are more likely to intersect the surgical field during deeper muscles splitting.

### Luminal diameter and tortuosity

Subset analysis of 30 subjects revealed a mean OA luminal diameter of 1.29 ± 0.39 mm at mid-mastoid level. This is consistent with the progressive diameter reduction reported by Alvernia et al. (2006), who measured 2.2–2.93 mm at origin and 1.9 ± 0.2 mm at the occipital groove exit, and by Yuan et al. (2023), who reported 1.65 mm at the superior nuchal line. The lack of significant difference in diameter between primary types suggests that the classification captures topographic relationships independent of vessel calibre.

Tortuosity was highly prevalent: 88.3% of arteries demonstrated some degree of tortuosity, and 20% showed severe tortuosity (kink or coil). This finding has two important implications. First, surgeons should anticipate tortuous OA morphology in the majority of cases when planning harvest or mobilisation. Second, the high prevalence of tortuosity provides methodological justification for our categorical rather than metric approach to OA-sulcus classification: measuring the distance between a tortuous vessel and the sulcus would yield values that vary continuously along the vessel’s course, rendering single-point measurements arbitrary and poorly reproducible.

Notably, Type 1 (intrasulcal) arteries demonstrated the highest rate of straight morphology (22.6%), while Types 2 and 3 showed universal tortuosity. Combined with the finding that Type 1 arteries have significantly lower infraatlantal rates, this suggests that the intrasulcal course may represent a “favourable” anatomical configuration characterized by both predictable location (within the sulcus, supraatlantal) and straighter morphology.

### Future directions: image-guided navigation in the lateral skull base

The detailed CTA-based characterisation of OA topography presented in this study may ultimately contribute to emerging image-guided surgical navigation systems for the lateral skull base. Trans-mastoid and craniocervical approaches require navigating through millimetre-scale surgical corridors where misnavigation can result in iatrogenic hearing loss, dizziness, and facial paralysis. Current surgical navigation systems employing optical or electromagnetic tracking methodologies are infrequently used in the lateral skull base due to the additional equipment required, the need to maintain line of sight of tracked instruments, electromagnetic interference, and the high accuracy requirements of lateral skull base surgery [3].

Recent developments in trackerless navigation using simultaneous localisation and mapping (SLAM) algorithms offer a promising alternative. It was demonstrated that SLAM-based surgical navigation using 3D exoscopy can achieve mean registration errors of 1.43 ± 0.49 mm and mean reconstruction errors of 0.72 ± 0.32 mm in deceased-donor models of trans-mastoid surgery, with accuracy approaching the submillimetric goal for lateral skull base surgery [3]. The system generates 3D surface models of the operative field from stereoscopic video and coregisters these with volumetric CT models using corresponding anatomical features, enabling continuous surgical navigation without external tracking equipment. Importantly, navigation accuracy is maintained throughout surgical dissection (exoscope-exoscope surface-surface registration error 1.72 ± 0.59 mm) and during switches between exoscopy and endoscopy (endoscope-exoscope surface-surface registration error 1.49 ± 0.61 mm) [3]. Integration of detailed OA topographic data, such as the classification system presented here, into such navigation platforms could further enhance surgical planning and intraoperative decision-making for procedures involving the suboccipital region.

### Limitations

Several limitations should be acknowledged. First, this is a single-centre study with a Romanian population, and the findings may not be generalisable to other ethnic groups. Second, CTA provides static imaging that may not fully capture dynamic intraoperative conditions such as soft-tissue retraction and positional changes. Third, the study did not assess OA calibre, which is an important determinant of suitability for bypass procedures. Fourth, correlation with intraoperative findings was not performed. Future studies incorporating direct surgical validation and measurement of OA dimensions would strengthen the clinical applicability of this classification. Fifth, OA tortuosity was assessed qualitatively rather than with quantitative indices. Standardised tortuosity metrics (e.g., distance factor, sum of angles) would require dedicated measurement protocols and therefore fall outside the scope of this study. Sixth, CTA provides excellent visualisation of bone-vessel relationships but has limited ability to distinguish individual muscle layers in the suboccipital region. The classification system was therefore intentionally designed around bony landmarks (occipital sulcus, mastoid process, atlas transverse process), which are consistently visualised on CTA and serve as reliable intraoperative reference points. Cadaveric or MRI-based studies would be required to correlate our CTA-based classification with the precise muscular planes traversed by the OA.

Seventh, OA-to-sulcus distance was not measured. This was a deliberate methodological choice: the occipital sulcus exhibits variable morphology (canal, groove, or poorly defined), and 88.3% of arteries in our subset analysis showed tortuosity. The distance between a tortuous vessel and an inconstantly expressed bony landmark would vary continuously along the vessel’s course, rendering any single measurement arbitrary. Our categorical classification captures the clinically relevant relationship – whether the sulcus reliably predicts OA location – without this methodological ambiguity.

## Data Availability

The datasets used and analyzed during the current study are available from the corresponding author upon reasonable request.
